# The ACTonHEART study: rationale and design of a randomized controlled clinical trial comparing a brief intervention based on Acceptance and Commitment Therapy to usual secondary prevention care of coronary heart disease

**DOI:** 10.1186/1477-7525-12-22

**Published:** 2014-02-19

**Authors:** Chiara AM Spatola, Gian Mauro Manzoni, Gianluca Castelnuovo, Gabriella Malfatto, Mario Facchini, Christina L Goodwin, Matteo Baruffi, Enrico Molinari

**Affiliations:** 1 Istituto Auxologico Italiano IRCCS, Psychology Research Laboratory, Milan, Italy; 2 Department of Psychology, Catholic University of Milan, Milan, Italy; 3 Cardiology Division, Ospedale San Luca, Istituto Auxologico Italiano IRCCS, Milano, Italy; 4 Department of Psychology, The Ohio State University, Columbus, OH, USA

**Keywords:** Ischemic heart disease, Modifiable cardiovascular risk factors, Adherence, Psychological well-being, Quality of life, Acceptance and Commitment Therapy, Behavior modification, Randomized clinical trial

## Abstract

**Background:**

Modifiable risk factors, including life-style habits and psychological variables, have been increasingly demonstrated to have an important role in influencing morbidity and mortality in cardiovascular patients, and to account for approximately 90% of the population risk for cardiac events.

Acceptance and Commitment Therapy (ACT) has shown effectiveness in promoting healthy behaviors, and improving psychological well-being in patients with chronic physical conditions. Moreover, a first application of an acceptance-based program in cardiac patients has revealed high treatment satisfaction and initial evidences of effectiveness in increasing heart-healthy behaviour. However, no clinical trial to date has evaluated the efficacy of an acceptance-based program for the modification of cardiovascular risk factors and the improvement of psychological well-being, compared to usual secondary prevention care.

**Methods:**

Approximately 168 patients will be recruited from an outpatient cardiac rehabilitation unit and randomly assigned to receive usual care or usual care + a brief ACT-based intervention. The ACT group will be administered five group therapy sessions integrating educational topics on heart-healthy behaviours with acceptance and mindfulness skills. Participants will be assessed at baseline, six weeks later (post treatment for the ACT condition), at six and twelve months follow-up.

A partially-nested design will be used to balance effects due to clustering of participants into small therapy groups. Primary outcome measures will include biological indicators of cardiovascular risk and self-reported psychological well-being. Treatment effects will be tested via multilevel modeling after which the mediational role of psychological flexibility will be evaluated.

**Discussion:**

The ACTonHEART study is the first randomized clinical trial designed to evaluate the efficacy of a brief group-administered, ACT-based program to promote health behavior change and psychological well-being among cardiac patients. Results will address the effectiveness of a brief treatment created to simultaneously impact multiple cardiovascular risk factors. Conducted in the context of clinical practice, this trial will potentially offer empirical support to alternative interventions to improve quality of life and reduce mortality and morbidity rates among cardiac patients.

**Trial registration:**

clinicaltrials.gov/ (NCT01909102)

## Background

It has been demonstrated that modifiable risk factors play an important and influential role in the morbidity and mortality of cardiovascular patients [1-3] and account for more than 90% of the population risk for acute myocardial infarction (MI) [4]. A recent meta-analysis by Janssen and colleagues [5], which analyzed 23 randomized trials of primary and secondary care programs developed from 1999-2009, indicated that life-style modification programs may improve risk factors and related life-style behaviors, and reduce mortality, cardiac readmission, and non-fatal infarctions. However, research has shown poor long-term adherence to recommended behaviors among cardiac patients even after the application of life-style intervention programs [1,6-8]. Moreover, the majority of successful programs were both costly and labor intensive, utilizing numerous sessions and lengthy intervention periods [5,9-13]. These time- and cost-consuming interventions reduce the adoptability of the program to applied medical settings.

In the past, secondary prevention programs were almost exclusively focused on exercise. However, in order to address the multiplicity of cardiovascular risk factors, secondary prevention programs should provide integrated behavioral care including exercise, health education, lifestyle counseling, and psychological treatment to address a wide range of modifiable risk factors such as co-morbid presentations of both heart disease and depression for example [14].

As such, it is important that other interventions are developed and evaluated to address this shortcoming. ACT [15], a “third wave” behavioral and cognitive therapy, has a growing empirical base demonstrating its efficacy for a wide range of conditions [16,17]. ACT has been modified and empirically evaluated in several chronic physical illnesses with varying treatment lengths (3-hour workshop to 3-week retreats) [18]. ACT aims to maximize psychological flexibility, which is the ability to be fully present in each moment and to persist or change behavior in the service of personal values [16].

Applications of acceptance-based interventions have proven efficacious in fostering health behaviors, such as weight management [19,20], physical activity practice [21], and smoking cessation [22]. This model has been successfully adapted to improve adherence to medical prescriptions and adaptive functioning among patients with chronic physical conditions, such as diabetes [23], epilepsy [24,25], multiple sclerosis [26], chronic pain [27], and HIV disease [28]. All of these ACT-based interventions had short treatment durations, and were often administered in a group setting. A large scale, randomized trial investigating the effectiveness of an acceptance-based intervention to improve the health and quality of life among cardiac patients has yet to be conducted. However, a pilot study by Goodwin and colleagues [29] evaluated an application of an acceptance-based program for cardiac patients and provided initial evidence for effectively increasing heart-healthy behaviours. Moreover, participants in this study were highly satisfied with the intervention. In particular, results showed large improvements in diet and moderate increases in physical activity from pre- to post-treatment. However, given the absence of a control group and the small sample size of this initial investigation, large-scale randomized clinical trials (RCT) are needed to test the efficacy of ACT-based interventions with cardiac patients.

### Rationale for applying ACT to promote life-style changes and adherence among cardiac patients

ACT is mainly focused on improving psychological flexibility by fostering the ability to behave in a values-driven manner. Traditional cognitive behavior therapy attempts to dispute, change, and restructure the content of negative thoughts to reduce consequent psychological discomfort. Rather than modifying the content of thoughts, ACT attempts to change the relationship between the person and his own thoughts and feelings [30]. In the ACT model, experiential avoidance is defined as the attempt to alter the form, frequency, or situational sensitivity of difficult internal experiences such as thoughts, emotions, and physical sensations often resulting in increased distress [31]. Experiential avoidance may explain the difficulty patients experience when faced with behavior change despite knowledge of medical risks.

Adopting and maintaining a healthy life-style often entails distressing physical sensations, thoughts, and feelings; thus individuals with a low ability to tolerate distress might be less likely adopt and maintain behavioural changes. For example, changes in diet often require compromises in portion-size and taste [32-34], exercise is associated with physical discomfort and exercise-anxiety [21], and abstaining from nicotine is associated with cravings [22,35]. Moreover, lower medication adherence has been reported among HIV-positive patients avoiding distress [36]. Across domains, decreases in experiential avoidance may increase adherence to health-behaviours.

Another ACT process useful in explaining behavior change is the connection with key personal values, defined as long-term desired qualities of living [16]. When an individual loses contact with his life value, he may sacrifice long-term behavioral goals and values (e.g., adopting a healthy lifestyle) in the service of decreasing his very salient short-term goals (e.g., reducing present discomfort). Short- and long-term distress tolerance is necessary to adopt healthy (but strenuous) lifestyle changes.

Given ACT’s ability to address core theoretical processes proposed to contribute to behavior change, ACT appears to be particularly promising for increasing adherence to low-risk lifestyles among cardiac patients (e.g., low fat diet, smoking cessation, medication adherence)—a fundamental, but difficult goal to be attained.

### Rationale for promoting psychological well-being among cardiac patients using ACT

Given increasing evidence to suggest a causal association between mood, stress, and the development and prognosis of coronary heart disease [37-39], the management of psychological distress should represent an important goal of secondary prevention programs.

In the last decade many studies on psychological flexibility indicated an inverse association with a wide range of psychological outcome measures (e.g., depression, anxiety, job induced tension, worries, pain) and a positive association with quality of life, perceived health, and positive emotional experiences with medium effect-sizes [16,40-47]. Moreover, recent research on the ACT model suggests that psychological flexibility mediates the effects of adaptive coping strategies on emotional and psychological well-being [48,49].

Research to date supports the utility of ACT-based interventions to modify health-behaviours and reduce psychological distress. Thus, we propose that an ACT-based intervention maybe useful for improving the physical and psychological health of cardiac patients.

#### Study objectives and hypotheses

To the best of our knowledge this will be the first randomized controlled trial to evaluate the efficacy of ACT in cardiac patients. Thus, the main aim of this research is to examine the efficacy of a manualized ACT-group therapy program for preventing the long-term deterioration of several risk factors and lifestyle behaviors and for improving psychological well-being and quality of life in patients with IHD over a 12-months follow-up period. In order to reflect Medical Research Council recommended guidelines [50] as well as the multi-factorial nature of the intervention, a number of primary outcome measures will be considered.

Moreover, the study is aimed at exploring the mechanisms of change surrounding the intervention with increased psychological flexibility as a possible critical mediator of intervention effects.

Specifically, the current study is designed to test the following hypotheses:

Primary hypothesis: The proposed ACT intervention will be associated with significantly better achievement, compared to the usual care, in at least two of the following primary outcome measures: LDL-cholesterol, resting systolic blood pressure, body mass index, and psychological well-being measured by the Psychological General Well-Being (PGWB) Index.

As no RCT was available on ACT-based interventions for cardiac patients, at the time of the study design we were not able to predict the modification of these four outcome variables as effect of the intervention.

Otherwise, expected levels of change on some of these indicators can be projected for participants in the usual care condition based on the GOSPEL study [51], a recent Italian survey on a similar population. Results from this study showed time-related worsening of the management of cardiac risk factors at 12-months follow-up. In particular, in the usual care condition, the following mean changes from baseline to 12-months follow-up were observed: an increase of 0.4 mg/dl in LDL cholesterol levels, an increase of 0.4 Kg/m^2^ in BMI and a 15.2% decrease in the rate of patients at target for resting systolic blood pressure [51].

We hypothesize that participants in the usual care group will show mean changes in the aforementioned variables comparable to those founded in the GOSPEL study, while patients in ACT condition will achieve better results on these primary outcomes with a medium between-group effect size (a standardized mean difference between mean changes of at least 0.3 standard deviations is expected).

*Secondary hypotheses:* The intervention will also improve a number of secondary outcomes: *1.* adherence to a heart-healthy lifestyle (increased physical activity; improved dietary patterns, smoking status, medication adherence); 2. perceived health-related quality of life; 3. other biological indicators of cardiovascular risk (exercise capability during an incremental bicycle stress test, lipid profile, glycosylated haemoglobin level, and waist to hip ratio).

*Mediation hypotheses:* The intervention will improve levels of psychological flexibility and the change in this variable will be associated with change in outcome variables.

## Methods

### Study design

This is a prospective randomized controlled clinical trial. It is a two-arm, partially nested design with three follow-up assessments (at the end of the treatment period, 6 and 12 months). Participants in the ACTonHEART condition will be provided usual care plus an ACT-based group program while those in the control condition will receive standard usual medical care. Indeed, according to the Medical Research Council guidelines [50] the ‘usual treatment’ may be more appropriate than a placebo condition in the case of trials evaluating complex interventions.

The study protocol was approved by The Istituto Auxologico Ethics Committee (2007000303).

#### Usual general practice care

Participants in both conditions will receive a standardized outpatient CR program designed in accordance to the best EU practice [14]. The program is individually tailored and conducted by a multi-disciplinary team. The CR physician is responsible for prescribing medication according to guidelines. The program has an average duration of 6-weeks and includes exercise training, psycho-education, and behavioral counselling by the CR team or a private practitioner. A letter will be sent to family physicians recommending secondary prevention goals. After the standard care period, patients will be asked to report to the hospital to complete a 6-month and 12-month assessment consisting of laboratory and exercise tests. After each assessment, a copy of said results will be forwarded to the participant’s family physician, who will continue to provide on-going treatment and medical advice as needed. In addition to standard care, the ACTonHEART group will receive the ACT intervention.

#### The ACTonHEART intervention

Participants randomly assigned to the ACTonHEART condition will complete five 90-minutes sessions over the course of 6-weeks based on ACT with the aim of positively modifying health-related behaviors and improving psychological health. The program will be developed on the basis of ACT-consistent protocols adapted from a) Goodwin and colleagues’ Acceptance Based Behavior Therapy (ABBT) for cardiac patients [29] and b) Acceptance-based treatment for the self-management of diabetes [23]. The program integrates educational topics on heart healthy behaviours with mindfulness and acceptance training, address difficult thoughts and feelings, clarification of health-related values, and behavioral commitment. The intervention will be administered by two interventionists (ACT therapist and doctoral-level student) in treatment groups of 8 participants.

Sessions 1-4 of the intervention will be delivered weekly, followed by a two week break between session 4 and 5. The content of each group-session will be structurally flexible, and include both didactic and experiential and metaphorical examples.

The sessions will be conducted at the cardiac rehabilitation gymnasium and each of them will last 90 minutes.

The ACTonHEART intervention will include the following four components:

*Cardiac education,* which consists of providing information about recommended diet, exercise, and the effects of risky behaviors such as smoking and poor adherence to medication. Additionally, specific behavioral techniques will be presented to address some of the common barriers to adoption of a heart-healthy lifestyle. Moreover, each session will include group problem-solving to address barriers to meeting individual behavioral goals.

*Mindfulness*, refers to engaging in one’s present moment experience and adopting an open and curious attitude instead of struggling with moment, even if the moment is unpleasant [15,52]. Additionally, mindfulness aims to increase self-awareness by noticing all internal experiences (positive and negative) in the moment. Previous control-based attempts to cope with cardiovascular disease, such as avoidance of disease cues, unpleasant thoughts and sensations, will be explored to demonstrate the ineffectiveness of control when pursuing a meaningful life. Participants will be encouraged to defuse from thoughts and feelings by turning attention toward the ‘noticing-self’ (termed defusion) instead of becoming attached to thoughts and maneuvering through life on ‘auto-pilot’.

*Acceptance and willingness*, involves the active awareness and embrace of difficult private internal experiences without unnecessary attempts to control or avoid unpleasant emotions. Acceptance is an alternative to experiential avoidance. Participants will be taught to increase adherence to a heart-healthy lifestyle by increasing willingness to experience distressing sensations and urges moment to moment (i.e., acceptance) while engaging in health behaviors. By encouraging participants to live in accordance with their values, participants can engage in meaningful/pleasurable activities despite experiencing unwanted emotions/sensations.

*Values, goals and committed action*. Identification of participant values and related behavioural goals will increase engagement in values-driven behavior, or committed action. Potential barriers to adopting value-driven behaviours and maintaining committed action in the long-term will be explored and addressed through metaphors and experiential techniques.

### Participants, setting, and assignment

All participants referred to the outpatient CR unit at S. Luca Hospital, Istituto Auxologico Italiano, Italy, will be invited to participate in the study. Patients who wish to participate will be screened for inclusion and exclusion criteria.

Patients who will meet the following inclusion criteria will be eligible for the study:

a) A definite diagnosis of IHD including recent (< 8 weeks) acute MI, acute coronary syndrome, surgical revascularization (Coronary Artery By-pass Surgery)

b) Major modifiable cardiovascular risk factors assessed using the INTERHEART Modifiable Risk Score [53]; higher scores indicate greater risk. Only subjects with a full score > 9 will be included in the study

c) Age between 18 and 70 years

d) Fluency in spoken and written Italian language

e) Expression of written informed consent

Exclusion criteria for the study are: a) severe psychiatric disorders according to DSM-IV TR criteria [54] b) mental incapacity to participate in the programme (e.g. cognitive impairment).

### Assessment procedure

Participants will be asked to complete all study measures before randomization. Follow-up repeated measures assessment will be completed on all study participants six weeks later (post treatment), and at 6- and 12-month follow-up.

Demographic data such as gender, age, marital status, level of education, and employment status will be assessed in order to control for these potential confounders.

Medical data such as recurrence of cardiac events and additional diagnoses (e.g. diabetes) that may confound results will be retrieved from clinical records. Furthermore, a listing of participants’ current medications and dosages at each time point will be used to assess the degree to which the intervention is affecting change above and beyond medication effects.

Participants in both groups will provide a history of any psychological treatment or counselling to assess potential familiarity with mindfulness- and acceptance-based therapies.

A range of physiological and psychological outcome measures will assess the following: modifiable cardiovascular risk factors, adherence to medication and recommended life-style, and psychological well-being and quality of life.

Adjusted change scores for all the outcome measures will be calculated in order to assess the interventions ability to produce changes over time.

#### Biological indicators of risk

Laboratory analysis will include fasting total cholesterol (mg/dl), HDL and LDL cholesterol (mg/dl), triglycerides (mg/dl), and glycosylated haemoglobin (%). Each blood sample will be analyzed by a certified laboratory.

Resting blood pressure will be assessed via an aneroid sphygmomanometer. Exercise capability will be considered the maximal power (Watts) reached during an incremental bicycle stress test. Moreover, an additional index of oxygen consumption, the double product, will be calculated as a heart rate x systolic blood pressure product.

#### Psychological well-being

Psychological health will be measured by the Psychological General Well-Being Index [55], validated in Italian [56]. The PGWB is a self-administered questionnaire composed by six sub-scales: anxiety, depression, positive well-being, self-control, general health and vitality. Higher scores indicate better health.

#### Quality of life

Health-related quality of life will be assessed by the SF36 questionnaire [57]. It is a widely used self-report instrument validated in Italian [58], which is composed by eight sub-scales: physical function, physical role, general health, social function, experience of pain, mental health, and emotional role and vitality. Physical component summary (PCS) and mental component summary (MCS) measures [59] will be used inlieu of the eight dimensions of the SF-36 to reduce the number of statistical comparisons and type 2 error. Higher scores indicate better quality of life.

#### Dietary habits

The Mediterranean diet has received support for the primary prevention of acute coronary syndrome [60,61]. Results from the CARDIO2000 study [62] showed an inverse association of such dietary pattern with serum lipids, blood pressures, inflammation, and coagulation markers related to cardiovascular disease. Moreover, individuals with higher scores of adherence to the Mediterranean diet were less likely to have acute coronary syndrome [60]. Since several studies have suggested that dietary patterns are more predictive of disease risk and mortality than individual foods or nutrients [63,64], an overall diet assessment will be assessed through the Mediterranean Diet Score [62], based on inherent characteristics of the Mediterranean diet. Questions focus on the frequency of usual consumption of 11 main components of the Mediterranean diet and each item is scored on an ordinal scale from 0 to 5. Scores are summed to obtain a total score ranging from 0 to 55.

#### Exercise

Participants’ physical activity levels will be measured by The International Physical Activity Questionnaire (IPAQ) [65], a self-report questionnaire that assesses the number of days per week and the number of times per day an individual spends doing specific activities. The questionnaire consists of 5 categories: job-related physical activity, transportation physical activity, housework, household tasks including family-care, recreation, sport and leisure time, and time spent sitting. In order for an activity to be reported, a minimum of 10 continuous minutes must be spent doing the activity.

#### Smoking status

Self-reports of smoking status were collected from participants at the end of treatment and at 6- and 12-months follow-up. The actual smokers will be also asked to report the average number of cigarettes smoked per day. The 7-day point prevalence abstinence rate at each assessment point will be used to assess the change of smoking status over time.

#### Adherence to medication

Adherence to pharmacological treatment will be measured by the Morisky Medical Adherence scale-8 items (MMAS) [66] that was developed from a previously validated four item scale [67] and supplemented with additional items addressing 1) the factors that may affect adherence 2) the circumstances surrounding adherence behavior and 3) problems with the complexity of the medical regimen The MMAS has a dichotomous response category for each item and a 5-point Likert response for the last item (“How often do you have difficulty remembering to take all your medication?”). Higher scores indicate lower adherence. The measure has proven to be reliable (α = 0.83) and has shown good concurrent and predictive validity [66]. The Italian version of the scale has been recently validated [68].

#### Psychological inflexibility

Psychological inflexibility will be measured through the Acceptance and Action Questionnaire-II [69]. The Italian version of the questionnaire has shown good reliability (α = 0.83) and high convergent validity [70]. Furthermore, disease-specific psychological inflexibility will be measured using the Cardiovascular Disease Acceptance and Action Questionnaire (CVD-AAQ), created for the proposed investigation. The CVD-AAQ was created by refocusing items from the AAQ-II [69] toward cardiovascular disease. The CVD-AAQ measures acceptance of thoughts and feelings related to cardio-vascular illness and the degree to which they interfere with valued action. It consists of 7 Likert-type items and showed good reliability in a preliminary study with 258 cardiac patients. Correlations with the AAQ-II [69] provide preliminary support for the convergent validity of the CVD-AAQ. Moreover, CVD-specific in flexibility is inversely associated with measures of psychological well-being and medication adherence.

### Sample size

The primary outcome variables of this study are the mean changes on the following indicators: LDL-cholesterol, resting systolic blood pressure, body mass index, and psychological well-being measured by the PGW.

As outlined above, at the time of the study’s design, we were unable to project the level of improvement on these outcome measures as effect of the ACT intervention over a 12 months follow-up period, since no data on cardiac patients were available.

Hypothesizing a medium effect size of the ACT intervention (in particular a standardized effect of at least 0.3 standard deviations) the following between-group differences on mean changes from baseline to 12-month follow-up are expected: 8.8 mg/dl in LDL cholesterol, 4 mmHg on systolic resting blood pressure and 1.3 kg/m^2^ in BMI. According to general guidelines, a power level of .80 is considered adequate to detect treatment effects [71]. In accordance with deJong and colleagues [72], a clustered design will require 160 participants to detect medium treatment effects with a statistical power of 0.8 and a significance level of .05.

Assuming a drop-out rate of <5% (as found in the GOSPEL study [51]), a sample size of 168 participants was finally defined, resulting in 84 participants being randomly assigned to each group.

Data on “lost to follow-up” participants will be analyzed to identify any relevant characteristics that may differ from the remainder of the study group. All data for the primary and secondary outcome measures will be analyzed using an intention-to-treat analysis such that participants will remain in their randomly assigned groups at final analysis, regardless of actual participation within such group.

### Randomization procedure and blinding

After the baseline assessment, participants are randomized to either ACTonHEART or usual care with the condition assignment determined via a computer generated algorithm. A block randomization scheme is employed, balancing gender and baseline impact of modifiable cardiovascular risk factors assessed using the INTERHEART Modifiable Risk Score (highest tertile vs. middle tertile). Participants allocated to the ACT condition are then scheduled into treatment groups. Randomization and follow-up assessments will be administered by independent practitioners blind to condition assignment and research hypotheses.

### Statistical analysis

Preliminary analyses will be conducted to test parametric statistical assumptions. If strong violations are detected, robust methods or data transformation will be applied. Outcome measures will be examined with intention-to treat analyses and analyses will be conducted on treatment completers.

Since participants in the ACT condition will be clustered into small therapy groups, the between-group variance should be taken into account. In fact, it has been shown that ignoring this level of nesting in the data can affect estimated variances and power to detect treatment and covariate effects [73], significantly inflate the Type I error rate and treatment effect size [74]. Thus, given the partially-nested design of this study, data will be analysed according to the guidelines suggested by Daniel J. Bauer and colleagues in their seminal paper [75]. Two-tailed tests with an adjusted critical alpha of 0.01 (0.05/4) will be conducted for examining the treatment effects on the four primary outcomes. Furthermore, on the basis of the best multilevel model structured in the primary analyses, the moderating effects of some putative covariates such as gender, age, level of education, marital status, cardiovascular disease status, and diabetes, will be explored and no adjustment to alpha will be applied. Beyond these analyses we also intend to explore the effects of ACT on measures of dietary habits and physical activity, smoking cessation, medication adherence and health-related quality of life, exercise capability during an incremental bicycle stress test, lipid profile, glycosylated haemoglobin level, and waist to hip ratio. The mediating role of psychological inflexibility will also be examined within the structured multilevel model. The MLwiN 2.21 sofware (Centre for Multilevel Modeling-CMM, University of Bristol) will be used for data analysis.

## Discussion

As shown by large epidemiological studies [76,77] cardiovascular risk can be described as multi-factorial and preventable because a greater percentage of the overall risk is comprised of modifiable risk factors [1-3]. Unhealthy life-styles, poor medication adherence, and psychological distress affect the prognosis of cardiac patients. ACT has proven efficacious at promoting and maintaining life-style changes and improving quality of life. Thus, it appears to be a promising method to improve quality of life and disease progression.

However there is a paucity of research examining treatment effects of ACT in the context of secondary prevention of IHD. Conducted in a clinical context, the ACTonHEART study will assess whether an ACT-based intervention (compared to usual care) can improve psychological health, and adherence to medical and behavioral regimens among a sample of patients diagnosed with IHD. ACTonHEART is developed in such a way that multiple risk behaviors are targeted at once. Although modification of each separate risk factor adds considerably to the standard care of cardiovascular patients, the majority of these patients have more than one risk factor. The compounded effects of multiple risk factors can be efficiently and effectively addressed with comprehensive interventions.

The ACTonHEART study is innovative in a number of ways. First, although ACT is becoming well-studied in the traditional clinical context, large-scale application to medical settings has not occurred. Second, the assessment framework incorporates indicators of both physical and psychosocial health. The inclusion of objective physiological outcome measures constitutes an aspect of innovation in ACT research. Last, the study will focus on mediation mechanisms underlying treatment success and presents possible links to systematic models on psychological flexibility, a key construct in ACT research.

Some aspects of the study design can limit recruitment and retention of participants. For example, the inclusion criterion of having an INTERHEART Modifiable Risk Score > 9 may slow recruitment. However, it is believed that floor effects and subsequent underestimation of treatment effects can be reduced by targeting individuals with adherence difficulties [78].

Among the major strengths of this study is the novelty of utilizing ACT to modify multiple cardiac risk factors with a brief intervention. This study is consistent with the call for prevention and health promotion research integrating psychosocial (e.g., depression) and behavioral (e.g., physical activity) risk factors [79]. Moreover, the inclusion of an EU standardized-care condition will provide important information pertaining to the added benefits of an ACT-based intervention and reduce inflation of treatment effects.

Another methodological strength of the study is the use of a multi-level model accounting for intraclass correlation due to clustering of patients in small therapy groups. Although several studies focuses on group therapy interventions, very few take control for this additional source of variance, potentially leading to significant bias.

This trial will be redacted following the CONSORT guidelines for non-pharmacological trials [80], and outcomes published in peer-reviewed clinical journals.

## Conclusions

To the best of the researcher’s knowledge, this study will be the first randomized controlled trial to examine the effectiveness of ACT to improve adherence to medical and behavioral prescription among cardiac patients. Conducted in the context of clinical practice, this trial will potentially offer empirical support to alternative interventions to improve quality of life and reduce mortality and morbidity rates among cardiac patients. Moreover, by virtue of its short duration, the program could potentially be utilized for health promotion and the primary prevention of CVD.

## Abbreviations

ACT: Acceptance and Commitment Therapy; ACTonHEART: Acceptance and Commitment Therapy on heart patients; IHD: Ischemic heart disease; CR: Cardiac rehabilitation; LDL: Low density lipoprotein; HDL: High-density lipoprotein; BMI: Body mass index; PGWB: Psychological General Well-Being.

## Competing interests

The authors declare that they have no competing interests.

## Authors’ contributions

CAMS conceived the study, planned its design and made substantial contribution to the manuscript drafting. GMM and CLG participated in the study design and contributed to the manuscript drafting. GC, EM, GM, MF and MB participated in the study design and helped to draft the manuscript. All authors read and approved the final manuscript.
